# Mechanical Properties and Damage Evolution of Shield Tunnel Spoil Solidified with Basalt Fiber-Reinforced Low-Carbon Cementitious Materials Under Drying–Wetting Cycles

**DOI:** 10.3390/ma19101920

**Published:** 2026-05-07

**Authors:** Yuhan Li, Henggen Zhang, Xujiayin Zhao

**Affiliations:** 1 School of Civil Engineering, Shandong University, Jinan 250002, China; yuhan_li@mail.sdu.edu.cn; 2 Shandong Key Laboratory of Technologies and Systems for Intelligent Construction Equipment, Shandong Jiaotong University, Jinan 250357, China; 3 School of Transportation and Civil Engineering, Shandong Jiaotong University, Jinan 250357, China; 4 Shandong Engineering Research Center of Marine Exploration and Conservation, Ocean University of China, Qingdao 266100, China; 5 Department of Civil, Environmental and Geomatic Engineering, University College London, London WC1E 6BT, UK; 15169857869@163.com

**Keywords:** shield tunnel spoil (STS), industrial solid wastes, basalt fibers (BF), drying–wetting (D-W) cycles, industrial computed tomography (CT)

## Abstract

This study aims to develop a green composite cementitious material (GCCM) by partially replacing cement with multiple industrial solid wastes and to further enhance its toughness by incorporating basalt fibers (BF) for the effective disposal of shield tunnel spoil (STS). The deterioration behavior of STS synergistically improved by GCCM and BF was systematically investigated under drying–wetting (D-W) cycles using unconfined compressive strength (UCS) tests, mass loss and P-wave velocity measurements, as well as industrial computed tomography (CT) and scanning electron microscopy (SEM). The results show that BF significantly improves the early-age strength and deformation toughness of STS, with an optimal UCS increase of about 13% at 0.45% BF. Although the mechanical properties of the specimens deteriorated with an increasing number of D-W cycles, the “bridging effect” of BF effectively inhibited the propagation and coalescence of cracks. Quantitative CT analysis further revealed that the addition of 1.00% BF reduced the pore volume (*V*_k_) and crack volume (*V*_l_) by 54.3% and 63.2%, respectively, after eight D-W cycles. The damage mechanism is primarily attributed to the loss of cementitious materials caused by water migration and the swelling–shrinkage stress of clay minerals. The three-dimensional (3D) network structure formed by BF, through its pull-out energy dissipation mechanism, effectively maintained the macro- and microstructural integrity of the material. This study highlights the novelty of combining GCCM with BF to enhance the long-term durability of STS, providing a theoretical basis and technical support for its green disposal and engineering application in complex environments.

## 1. Introduction

With the continuous advancement of global urbanization, the development of underground space resources has become an inevitable choice to alleviate surface pressure in cities [1]. The shield tunneling method, owing to its significant advantages such as high automation, fast construction speed, and minimal disturbance to the ground surface, has emerged as a core technology for the construction of urban deep infrastructure, including subways and river-crossing tunnels [2]. However, the shield tunneling process inevitably generates a substantial amount of STS. This spoil typically exhibits high moisture content, considerable clay particle content, and an extremely complex mineral composition, rendering its engineering properties highly unstable [3]. Traditional disposal methods, such as stockpiling and landfilling, not only occupy vast land resources and damage the original landscape but also readily trigger geotechnical instability hazards like landslides and debris flows under rainwater infiltration. Concurrently, these practices exert long-term negative impacts on the surrounding soil environment and ecological balance [4]. Therefore, achieving large-scale resource utilization of STS while ensuring engineering safety and ecological preservation has become a critical challenge urgently needing resolution in the fields of underground engineering and environmental geotechnics.

For the harmless treatment of STS, solidification/stabilization technology is widely recognized as one of the most effective approaches in both academia and engineering [5]. Although ordinary Portland cement (OPC) is extensively used in traditional soil solidification engineering, its high pollution, high energy consumption, and substantial CO_2_ emissions during production contradict the current concept of low-carbon construction [6,7]. Furthermore, the sole use of OPC for soil solidification is prone to volume shrinkage in the early service stage and brittle cracking in the later stage, making its durability difficult to guarantee [8]. Based on this, the partial or complete substitution of OPC with admixtures demonstrates significant economic and environmental potential [9,10,11]. Among these, ground granulated blast-furnace slag (GGBS), fly ash (FA), and flue gas desulfurization gypsum (FGDG) are commonly employed admixtures in soil solidification systems [12]. GGBS is rich in reactive glassy oxides, which can undergo hydration reactions upon activation in a high-alkalinity environment, generating high-strength and high-density calcium silicate hydrate (C-S-H) gel that provides core strength for the solidified soil [13]. FA, through its unique spherical microstructure, exerts a “micro-aggregate effect” that effectively fills the fine pores between soil particles [14]. Additionally, the reactive SiO_2_ and Al_2_O_3_ it contains can participate in secondary hydration reactions with calcium hydroxide (CH) produced by OPC hydration, generating additional cementitious products [15]. FGDG, acting as a sulfate activator, supplies ample ${\mathrm{SO}}_{4}^{2-}$ ions to regulate the formation of substantial amounts of needle-like ettringite (AFt). It leverages the unique expansion compensation effect of AFt to counteract the drying shrinkage of the matrix and significantly enhances structural compactness and impermeability through space filling [16]. CAP is rich in active aluminum phases, which synergize with ${\mathrm{SO}}_{4}^{2-}$ ions to accelerate the early nucleation and development of AFt. By forming numerous interwoven needle-like crystals, it establishes a micro-interlocking structure within the matrix, thereby significantly enhancing the structural strength during the initial and early stages of solidification [17].

Scholars worldwide have conducted extensive explorations on the partial substitution of cement with supplementary cementitious materials and functional components. Research generally indicates that optimizing the proportions of various reactive materials can fully stimulate the synergistic effects of physical filling and chemical activation within the system [18]. While substantially reducing the consumption of OPC clinker, the spatial interlocking achieved through multiple cementitious products generated by the hydration of multi-source components enables the solidified system to attain mechanical properties and durability that are comparable to or even better than those of pure OPC schemes. This provides a solid theoretical foundation for addressing the strength and stability challenges in the resource utilization of shield tunnel spoil [19].

During practical engineering service, solidified soil is exposed to drastic changes in the moisture field induced by alternating climate and groundwater table fluctuations, causing its microstructure to be severely tested by repeated D-W cycles [20]. The non-uniform swelling–shrinkage stress induced by D-W cycles often leads to the accumulation of physical damage within the solidified system. Existing research indicates that this damage evolution is essentially a kinetic process of microstructural reorganization and degradation of the cemented skeleton [21]. The repeated intrusion and migration of water trigger lattice expansion of clay minerals and chemical dissolution of the matrix, resulting in a significant decrease in the interfacial shear strength between the solidified products and soil particles [22]. Concurrently, if the tensile strain generated by drying shrinkage stress exceeds the tensile limit of the matrix, it drives the evolution of original micro-defects into penetrating fissures, forming preferential pathways for water infiltration, thereby accelerating performance deterioration [23]. For fillers with high clay content, such as STS, their significant hydrophilicity and swelling–shrinkage sensitivity make the durability challenge particularly prominent. To compensate for the inherent defects of the solidified matrix, such as high brittleness, low tensile strength, and poor deformation compatibility, the introduction of high-performance short fiber reinforcement materials has become a mainstream trend in civil engineering to enhance material toughness [24].

BF, as a natural inorganic mineral fiber derived from volcanic rock, exhibits great potential for improving the toughness of brittle matrices due to its excellent tensile strength, high elastic modulus, and chemical inertness in highly alkaline curing environments [25]. Previous researchers have conducted extensive studies on the reinforcement effect of BF, and the results generally indicate that BF can construct a complex three-dimensional randomly distributed reinforcement network within the soil. Through interfacial bonding force and mechanical interlocking between the fibers and the matrix, it exerts a “bridging” effect at the microscopic level, effectively limiting the initiation of micro-cracks [26]. At the macroscopic level, BF absorbs strain energy through a pull-out energy dissipation mechanism, which can significantly enhance the fracture energy and ductility of the solidified system [27]. The incorporation of an appropriate amount of fiber not only optimizes the stress–strain response of the solidified soil but also maintains the integrity of the structural skeleton under environmental loads [28].

Although previous researchers have made significant progress in the field of partial substitution of OPC with admixtures and fiber reinforcement, no systematic study has yet addressed the deterioration behavior and the associated 3D pore evolution mechanism of STS improved by multi-source solid waste components synergized with BF under complex D-W cycles, particularly regarding the coupled effects of cyclic moisture fluctuations on microstructural integrity. In view of this, this study systematically investigated the long-term durability performance of solidified STS through macro-micro multi-scale experimental approaches. In this study, a composite solidification system was constructed by partially substituting OPC with various industrial solid wastes, and different contents of BF were introduced to enhance the modification of STS. Through indoor D-W cycle tests, mechanical property tests, and multi-scale microstructural characterization, the intrinsic relationship between microstructural damage and macroscopic mechanical property evolution under environmental actions was deeply revealed. Unlike previous studies that focused on single admixtures or short-term performance under static or D-W conditions, this work systematically evaluates the long-term durability under cyclic moisture fluctuations using multi-scale characterization, combining industrial CT and SEM to quantify pore and crack evolution. The research findings can provide a scientific basis for technology selection in STS solidification treatment and its adaptability assessment under complex environmental conditions.

## 2. Materials and Methods

### 2.1. STS

**Figure 1:**
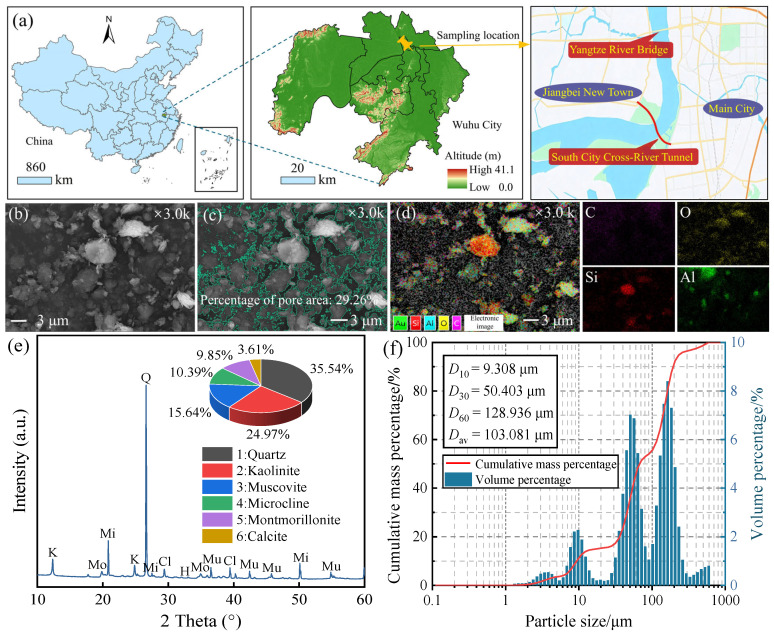
Laboratory experimental analysis results of intact STS: (**a**) sampling location of STS used in the experiment; (**b**) SEM image of the STS surface; (**c**) pore distribution on the STS surface; (**d**) elemental distribution on the STS surface; (**e**) XRD pattern of STS; (**f**) gradation curve of STS.

The STS used in this study was sampled from the Chengnan River-crossing tunnel project site in Wuhu City (see Figure 1a). To conduct an in-depth analysis of the physicochemical properties of STS, a series of characterization tests were carried out in the laboratory, including SEM, energy dispersive spectroscopy (EDS) at 3000× magnification, X-ray diffraction (XRD), and particle size distribution testing. XRD analysis was performed using a diffractometer equipped with a Cu–Kα radiation source. The X-ray tube was operated at a voltage of 40 kV and a current of 40 mA. Data were collected over a 2*θ* angular range of 10° to 60° with a step size of 0.02° and a counting time of 1 s per step. The obtained diffraction patterns were processed and analyzed using MDI Jade 6.0 software. Phase identification was carried out by comparing the measured patterns with the ICDD PDF-2 database. The specific characterization results are shown in Figure 1b–f. XRD analysis indicates that the main mineral components of STS include quartz (35.54%), kaolinite (24.97%), muscovite (15.64%), microcline (10.39%), montmorillonite (9.85%), and calcite (3.61%). The corresponding ICDD PDF reference cards used for phase identification are as follows: quartz (PDF#04-2085), kaolinite (PDF#00-2004), muscovite (PDF# 01-0929), microcline (PDF#00-0932), montmorillonite (PDF# 00-1499), and calcite (PDF#04-0489). Observation of the micromorphology revealed that the surface porosity of undisturbed STS is approximately 29.26%, and the elemental composition is predominantly C, O, Si, and Al. Table 1 summarizes the basic physical and mechanical parameters of STS. According to GB/T 50145-2007 [29], the STS collected from this site can be classified as silty sand.

### 2.2. GCCM and BF

This study developed a novel composite cementitious material, GCCM, for the solidification treatment of STS, with its design concept centered on achieving substantial substitution of traditional cement through the synergistic effects of multi-source industrial by-products. This solidification system is composed of OPC, GGBS, FA, FGDG, and the active component CAP. The mass ratio of GCCM is set as follows: OPC accounts for 50%, GGBS 25%, FA 10%, FGDG 10%, and CAP 5%. The chemical compositions of the raw materials used in GCCM are presented in Table 2, and the major clinker phase composition of OPC is presented in Table 3. The proportions of the raw materials in GCCM were determined through preliminary orthogonal experiments, which indicated that an OPC content of 50% ensures sufficient alkalinity for activating GGBS and FA, while the combined 10% FGDG and 5% CAP provides optimal ettringite formation without excessive expansion. The FA used in this study is classified as Class F with a pozzolanic activity index of 78% at 28 d. In this system, the initial hydration of OPC provides the necessary alkaline environment; GGBS, by virtue of its latent hydraulic property, rapidly reacts under alkali activation to form C-S-H gel; while FA further fills the pores of STS and optimizes the microstructure through its long-term pozzolanic effect. Furthermore, FGDG and CAP synergistically induce the formation of AFt crystals, significantly enhancing the macroscopic strength and volume stability of solidified STS through the interlocking filling and volume compensation effects of these crystals.

**Figure 2:**
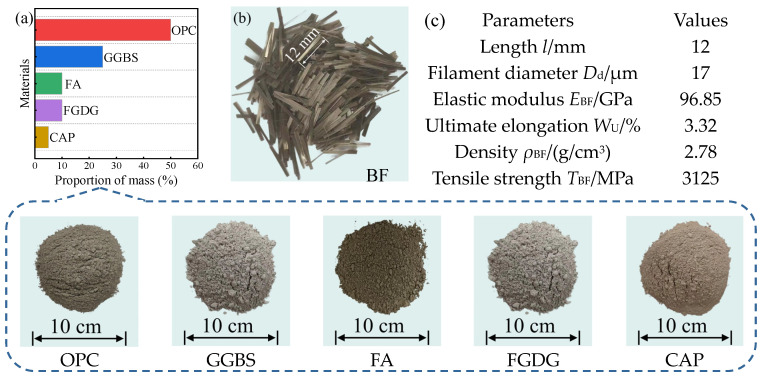
(**a**) Raw material components of GCCM, and (**b**) appearance and (**c**) physical and mechanical performance indicators of BF.

To enhance the crack resistance and toughness of solidified STS, this study introduced BF with a length of 12 mm as a reinforcement component. BF possesses excellent tensile properties and chemical stability, enabling it to construct a three-dimensional spatial network structure between GCCM hydration products and STS particles. This “reinforcement” effect can effectively distribute axial loads and delay the nucleation and propagation of micro-cracks during D-W cycles, thereby significantly improving the structural integrity of the stabilized STS. The raw material composition of GCCM and the specific physical and mechanical performance indicators of BF are detailed in Figure 2.

### 2.3. Experimental Procedure

**Figure 3:**
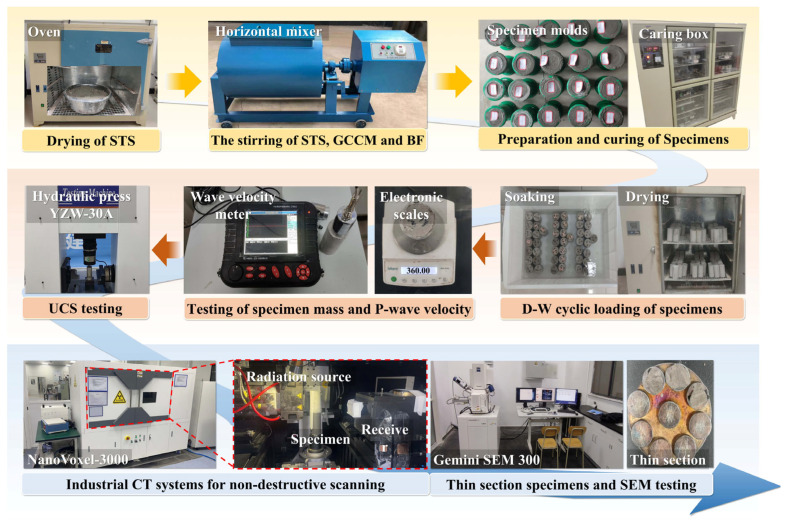
Experimental procedure and partial equipment diagrams.

The experimental procedure of this study is illustrated in Figure 3. During the specimen preparation stage, the content of the composite cementitious material GCCM was fixed at 20% of the dry mass of STS. The addition of BF was set at six gradients of 0.00%, 0.10%, 0.25%, 0.45%, 0.70%, and 1.00%, calculated as a percentage of the total mass of GCCM and STS. The BF contents were selected based on a preliminary range-finding study, which showed that contents below 0.10% had negligible reinforcement effects, while contents above 1.00% led to fiber balling and poor workability. The six gradients thus cover the full range from ineffective to excessive, allowing identification of the optimal dosage. The amount of water used during mixing was uniformly set at 35% of the total solid mass (the sum of GCCM, BF, and dry STS). The prepared specimens were cured under standard conditions for 28 d, after which D-W cycle tests were conducted. A single D-W cycle consisted of 24 h of forced water saturation and 24 h of oven drying (with the temperature set at 45 °C). A total of 0, 2, 5, 8, 12, and 16 cycles were designed for the experiment. The chosen D-W cycle conditions (24 h saturation followed by 24 h drying at 45 °C) were designed to represent typical field scenarios in shallow underground engineering, where seasonal rainfall and groundwater table fluctuations induce alternating saturation and evaporation. The drying temperature of 45 °C was selected to accelerate evaporation while avoiding thermal decomposition of hydration products (e.g., C-S-H, AFt), which typically occurs above 60 °C. This accelerated laboratory protocol provides a conservative and reproducible basis for evaluating long-term durability. The mass and P-wave velocity of the specimens were accurately measured and recorded in the dry state before and after each cycle. Upon completion of the preset D-W cycles, UCS tests were performed on the specimens in a dry state using a YZW-30A automatic hydraulic testing machine manufactured by Jinan Puye Electromechanical Technology Co., Ltd. (Jinan, China). After the mechanical tests, specimens from each BF content group that had undergone 8 D-W cycles were selected for non-destructive scanning using a NanoVoxel-3000 industrial CT system manufactured by Tianjin Sanying Precision Instruments Co., Ltd. (Tianjin, China). Threshold segmentation and 3D reconstruction techniques were employed to quantitatively extract the 3D spatial distribution characteristics of internal pores and cracks. In addition, specimens with a BF content of 0.45%—which exhibited the highest UCS among all fiber contents in the initial state (without D-W cycles)—were selected from each D-W cycle stage (0, 2, 5, 8, 12, and 16 cycles) for SEM observation using a GeminiSEM 300 manufactured by Carl Zeiss Microscopy GmbH (Jena, Germany), in order to investigate the influence of cycle number on micro-morphology. It should be noted that all specimens used for SEM and CT observations were taken from the samples that had already undergone UCS testing. In particular, the CT scanning was performed not only to quantify the pore volume but also to measure the size and spatial distribution of cracks generated during UCS loading. This experimental sequence ensures that the microstructural analysis directly corresponds to the mechanical performance of the same specimens.

## 3. Results and Analysis

### 3.1. Mass and V_p_

**Figure 4:**
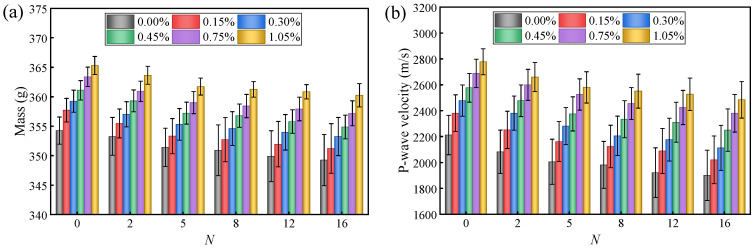
Changes in (**a**) mass and (**b**) P-wave velocity of solidified STS specimens after D-W cycles.

Figure 4 systematically illustrates the mass fluctuations and P-wave velocity evolution of solidified STS specimens with different BF contents during D-W cycles. In Figure 4 and subsequent figures, *N* represents the number of D-W cycles. The experimental results indicate that, in the initial state (without experiencing D-W cycles), the addition of BF had a significant positive effect on the compactness of the specimens. Compared with specimens without BF, the addition of 1.00% BF (by total solid mass of GCCM, STS, and BF) increased the specimen mass and P-wave velocity by 3.12% and 25.59%, respectively. This mass increase is attributed to the higher density of BF (2.78 g/cm^3^) and its pore-filling effect, which enhances the overall compactness of the specimens. This phenomenon reflects that BF can effectively fill the minor defects within the GCCM solidification system, and its 3D skeletal action optimizes the initial microstructure of the specimens, thereby enhancing the continuity of wave velocity propagation. With the increase in the number of D-W cycles, all specimens exhibited varying degrees of mass loss and P-wave velocity reduction. Taking the 0.45% BF content group as an example, after 16 cycles, its mass and P-wave velocity decreased by 1.73% and 12.69%, respectively. The mass loss of the specimens mainly originated from hydraulic erosion and physicochemical scouring during the cycles, which weakened the interfacial bonding between STS particles and GCCM cementitious products, leading to the detachment of surface mineral particles accompanied by increased porosity [30]. The deterioration of P-wave velocity further revealed the evolution of internal damage: the expansion of internal pore space and the coalescence of micro-cracks significantly increased the path tortuosity and energy scattering of ultrasonic wave propagation, resulting in a continuous decrease in wave velocity [31].

### 3.2. Mechanical Properties

**Figure 5:**
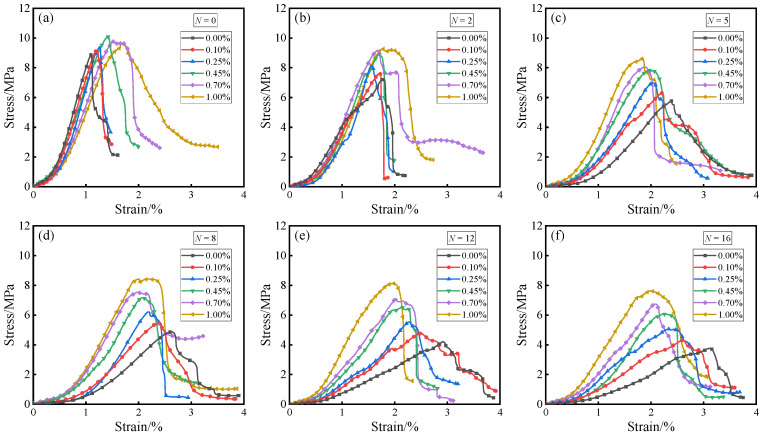
Stress–strain curves of solidified STS specimens after D-W cycles: (**a**) *N* = 0; (**b**) *N* = 2; (**c**) *N* = 5; (**d**) *N* = 8; (**e**) *N* = 12; (**f**) *N* = 16.

**Figure 6:**
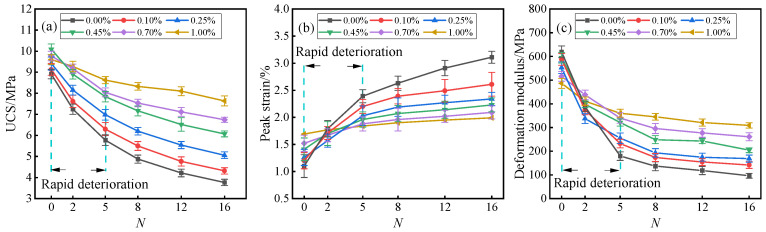
Changes in (**a**) UCS, (**b**) peak strain, and (**c**) deformation modulus of solidified STS specimens after D-W cycles.

Figure 5 and Figure 6 respectively illustrate the stress–strain response and the evolution of key mechanical indicators (UCS, peak strain, deformation modulus) of solidified STS specimens under uniaxial compression. As shown in Figure 5 and Figure 6, the incorporation of BF not only significantly enhanced the bearing capacity of the specimens but also induced a transition in failure mode from brittle to ductile. Without experiencing D-W cycles, the UCS of the specimens exhibited a trend of initially increasing and then decreasing with the increase in BF content, reaching a peak value of 10.09 MPa at a BF content of 0.45%, which was 13.24% higher than that of specimens with 0.00% BF. This enhancement effect is attributed to the “bridging” and “constraint” effects of BF within the GCCM gel matrix: benefiting from its high tensile modulus, BF can bear most of the tensile stress through interfacial bonding forces when spanning across micro-cracks, thereby effectively inhibiting the unstable propagation of cracks [32]. As the BF content increased from 0.00% to 1.00%, the peak strain of the specimens monotonically increased from 1.10% to 1.69%, while the deformation modulus correspondingly decreased from 619.15 MPa to 486.29 MPa. This phenomenon reveals that while BF enhances structural toughness, it also increases the deformability of the system to a certain extent due to the difference between its own modulus and that of the soil matrix [33]. Compared with previous studies on basalt fiber-reinforced cement soil, the compressive strength trend obtained in this study is consistent with reported findings. For example, Shu and Zhang [34] reported that adding 0.5% basalt fiber to cement soil achieved a compressive strength of 12.59 MPa. Chen et al. [35] reported that incorporating 0.4% BF into cement-stabilized expansive soil increased the compressive strength by 24.8% without D-W cycles. These comparisons further confirm the effectiveness of the GCCM-BF system in enhancing mechanical strength.

During the damage evolution process under D-W cycles, the mechanical properties of the specimens exhibited significant stage-dependent deterioration characteristics. Taking the 0.45% BF content group as an example, after 16 cycles, the UCS and deformation modulus sharply decreased by 39.84% and 65.65%, respectively, while the corresponding peak strain significantly increased by 58.16%. The strength attenuation law exhibited a characteristic of being rapid in the early stage and slow in the later stage, meaning that the main damage was concentrated within the first 5 cycles. This deterioration mechanism originates from the accumulation of micro-damage caused by repeated water ingress and egress: the non-uniform stress field generated by water absorption expansion and dehydration shrinkage leads to cracking and detachment of the GCCM matrix, weakening its effective bonding between STS particles [36].

However, a high content of BF demonstrated excellent resistance to deterioration, significantly slowing down the rate of strength degradation. BF played a role similar to three-dimensional reinforcement, and its large specific surface area provided ample interfacial frictional resistance, effectively limiting the relative slip between STS particles during axial compression [37]. Meanwhile, the presence of BF optimized the stress distribution, converting concentrated point loads into more uniform volumetric loads, thereby maintaining the relative stability of the internal micro-skeleton of the specimens under D-W cycles [38]. This fully demonstrates the great potential of the BF-GCCM composite system in enhancing the environmental durability for the resource utilization of STS.

### 3.3. Failure Mode Analysis

**Figure 7:**
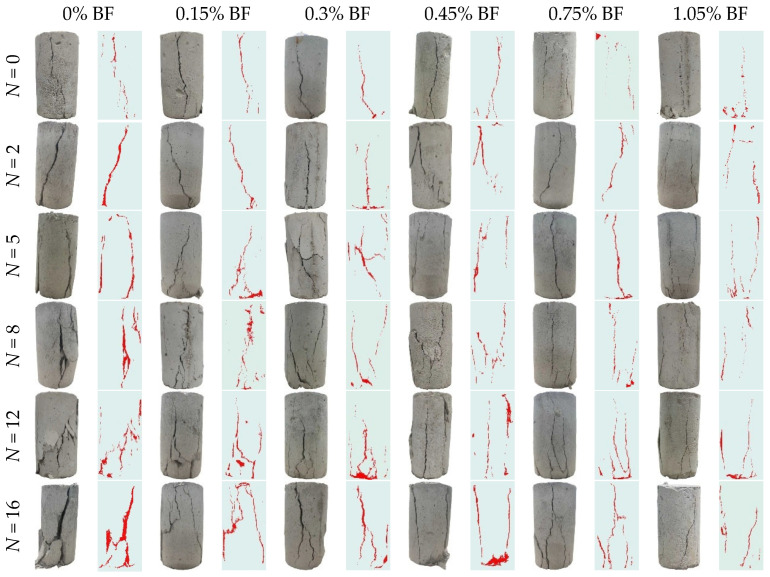
Failure modes of solidified STS specimens after D-W cycles.

Figure 7 presents the failure modes of solidified STS specimens after UCS tests. As shown in Figure 7, the failure modes of solidified STS specimens exhibited evolutionary characteristics driven by the coupled effects of BF content and the number of D-W cycles. Specimens that had not undergone D-W cycles displayed few surface cracks upon failure, with shear cracks being the predominant type. As the number of cycles increased, the failure mode gradually transitioned from single shear to tension-shear combined failure, with the crack network becoming more complex and fragmented. Under a high number of cycles, pronounced spalling areas tended to form at the bottom of the specimens. The addition of BF significantly inhibited crack development; under the same number of D-W cycles, specimens with higher BF content exhibited noticeably fewer surface cracks.

The transition in the failure mode of solidified STS specimens was primarily governed by the micro-damage evolution induced by D-W cycles. During the alternating D-W process, the repeated migration of water within the pores and the swelling–shrinkage effects of clay minerals led to the initiation of numerous randomly distributed micro-cracks inside the specimens [39]. Under axial loading, these micro-cracks evolved into stress concentration zones, inducing the nucleation and propagation of secondary cracks. The introduction of BF exerted significant “bridging” and “constraining” effects. By virtue of its high tensile modulus, BF spanned across crack surfaces and effectively shared the tensile stress borne by the matrix through interfacial friction and bonding forces, significantly limiting the unstable propagation rate of main cracks [40]. This physical reinforcement mechanism enhanced the deformation compatibility of the specimens during the process of damage accumulation, enabling them to maintain relatively high micro-skeletal integrity even after multiple D-W cycles, which macroscopically manifested as a delayed attenuation of compressive strength.

### 3.4. Microstructural Analysis

**Figure 8:**
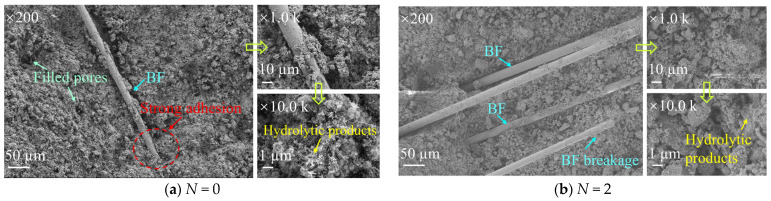

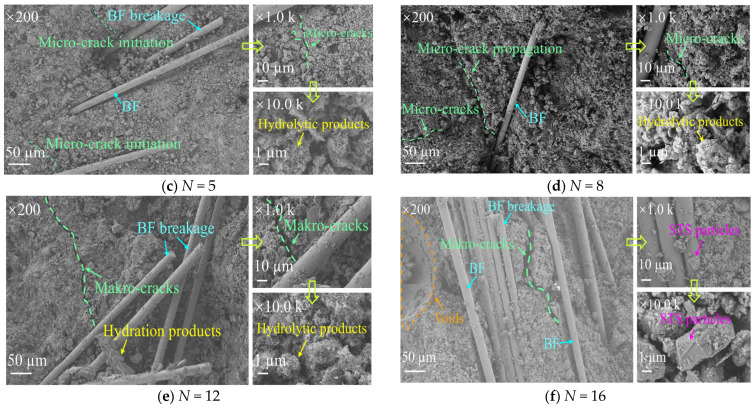
SEM images of solidified specimens after D-W cycles.

Figure 8 presents the microstructural evolution process of solidified STS specimens with 0.45% BF content as the number of D-W cycles increases. Before discussing the microstructural evolution, it should be clarified that due to the relatively low binder content (20% by dry mass of STS), hydration products such as C-S-H, AFt, and calcium aluminum silicate hydrate (C-A-S-H) were not sufficiently abundant to be clearly identified in the SEM images. Consequently, the SEM analysis in this study focuses on the qualitative observation of fiber morphology (deflection, separation, pull-out) and fiber-matrix interfacial bonding under different numbers of D-W cycles, rather than on detailed phase identification or EDS quantification of hydration products. In the initial specimens without D-W cycles (Figure 8a), the BF surfaces were deeply coated by STS particles and GCCM hydration products, exhibiting excellent interfacial bonding effect. This tight interfacial anchoring not only provided additional physical reinforcement force between particles but also served as the core mechanism for BF to enhance the macroscopic strength of the specimens. At this stage, flocculent C-S-H and calcium aluminate silicate hydrate (C-A-S-H) gels not only induced the agglomeration of fine particles but also significantly reduced the initial porosity of the matrix through space-filling effect. After experiencing 2 D-W cycles (Figure 8b), the microstructure began to show signs of performance deterioration. Affected by water erosion, the cementitious products on the BF surface underwent local peeling, the bonding constraint capacity of BF was weakened, and the matrix surface gradually presented a loose state. By the time of 5 D-W cycles (Figure 8c), under the action of water migration stress, discrete micro-cracks emerged inside the specimens. These micro-cracks became the main channels for subsequent water ingress and egress, accelerating the migration and loss of internal fine particles and soluble cementitious substances. As D-W cycles increased to 8 (Figure 8d), micro-cracks further expanded under non-uniform swelling–shrinkage stress, leading to the disintegration and refinement of the originally compact particle agglomerates. Entering the middle and late stages of cycles (12 cycles, Figure 8e), micro-defects evolved dramatically, with micro-cracks transforming into macro-cracks through cross-linking and coalescence, accompanied by the loss of a large number of hydration products, and pronounced dissolution pores formed on the specimen surface. When D-W cycles reached 16 (Figure 8f), the crack width significantly increased, and the surface presented a honeycomb-like pore structure due to severe particle spalling. Viewing the dynamic evolution process in Figure 8, it can be seen that D-W cycles continuously eroded the bonding layer on the BF surface, leading to the gradual loss of the fiber’s reinforcement effect with interface degradation, ultimately causing the integrity failure of the microstructure of the solidification system.

**Figure 9:**
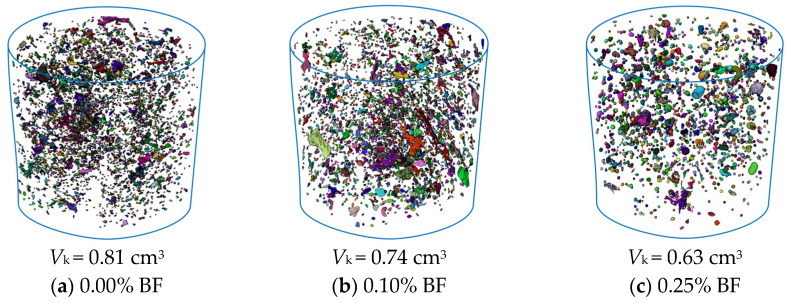

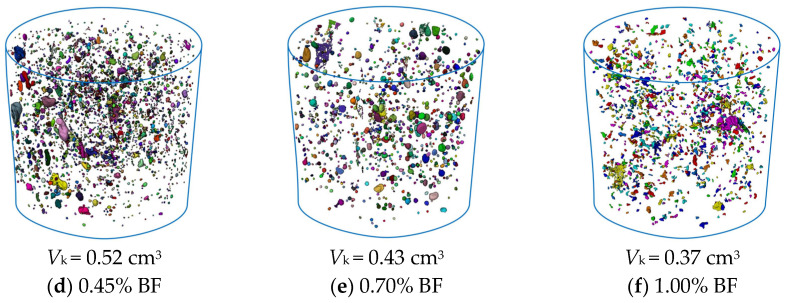
CT images of 3D pores of solidified STS specimens after D-W cycles.

**Figure 10:**
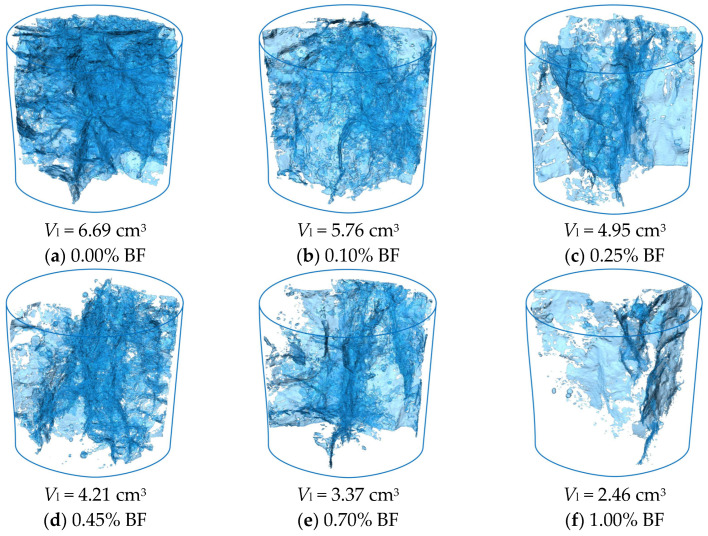
CT images of 3D cracks of solidified STS specimens after D-W cycles.

Figure 9 and Figure 10 quantitatively extract the 3D pore and crack topological characteristics of solidified STS specimens after 8 D-W cycles using threshold segmentation technique, where *V*_k_ and *V*_l_ represent the statistical volumes of pores and cracks, respectively. Quantitative analysis indicates that the introduction of BF plays a significant role in inhibiting structural damage induced by D-W cycles. As the BF content increased from 0.00% to 1.00%, the *V*_k_ of the specimens was reduced from 0.81 cm^3^ to 0.37 cm^3^ (a decrease of 54.32%), while *V*_l_ substantially decreased from 6.69 cm^3^ to 2.46 cm^3^ (a decrease of 63.23%). From the perspective of spatial distribution characteristics (Figure 9), the increase in BF significantly inhibited the initiation and development of micro-pores. This reveals that BF not only plays a filling and optimizing role for STS particles in physical space but also synergistically improves the compactness of the matrix with the GCCM solidification system. The introduction of BF promotes the formation of a more uniformly dispersed network structure of cementitious products within STS, reducing the formation of initial defects through the “micro-skeleton” effect, thereby enhancing the material’s resistance to D-W cycles from the source [41]. The 3D crack distribution (Figure 10) further confirms that BF effectively resists crack propagation. This crack resistance effect at the microscopic level directly manifests macroscopically as the maintenance of surface structural integrity of the specimens and the improvement of compressive strength, as shown in Figure 7.

## 4. Discussion

Based on the chemical reaction characteristics of each component in the GCCM system, it can be reasonably inferred that the main hydration products include C-S-H, C-A-S-H, and AFt. Specifically, OPC hydration provides Ca(OH)_2_ and C-S-H gel while establishing a high-alkalinity environment; GGBS undergoes secondary hydration under alkaline activation, generating additional C-S-H and C-A-S-H; the reactive SiO_2_ and Al_2_O_3_ in FA react with Ca(OH)_2_ to form C-S-H and C-A-S-H; SO_4_^2−^ supplied by FGDG reacts with aluminate phases to form AFt; and the reactive alumina in CAP further promotes the early formation of AFt.

### 4.1. Damage Mechanism Analysis of D-W Cycles

**Figure 11:**
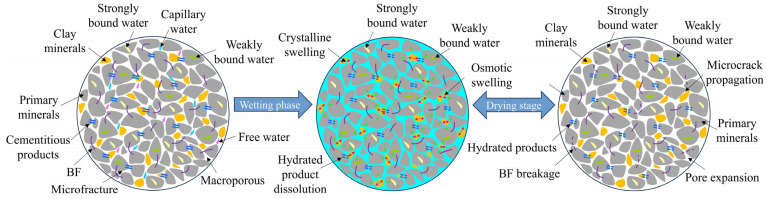
Structural damage evolution process of solidified STS specimens under D-W cycles.

Figure 11 illustrates the evolution mechanism of structural damage of solidified STS under D-W cycles. The clay minerals such as kaolinite and montmorillonite abundantly present in STS possess strong hydrophilicity, and this characteristic is the core factor driving the strength deterioration and pore swelling–shrinkage deformation of solidified specimens [42]. The expansion process of clay minerals upon contact with aqueous solution can be divided into two stages: crystalline swelling and osmotic swelling. In the crystalline swelling stage, water molecules enter the interlayer spaces of minerals, inducing cation hydration reactions, and the resulting interlayer repulsion forces compel the separation of crystal layers [43]. During the dehumidification (drying) process, the dissipation of water follows a kinetic gradient: free water in large pores vaporizes first at the surface driven by the internal and external vapor pressure difference; subsequently, capillary water in small pores, weakly bound water between particles, and interlayer water successively dissipate from the inside to the outside in the form of water vapor. Accompanied by the repeated migration of moisture during D-W cycles, hydraulic scouring leads to physical loss and particle migration of the cementitious materials generated by GCCM hydration, thereby causing radial expansion of pores and loosening of the matrix structure. Furthermore, the frequent cycles of swelling–shrinkage strain cause fatigue breaking of some BF, weakening the constraining effect of fibers on the matrix, and ultimately inducing the evolution of micro-cracks into macro-cracks, which manifests at the macroscopic level as the continuous deterioration of the compressive strength of the specimens.

### 4.2. Relationship Characteristics of Mechanical Parameters

**Figure 12:**
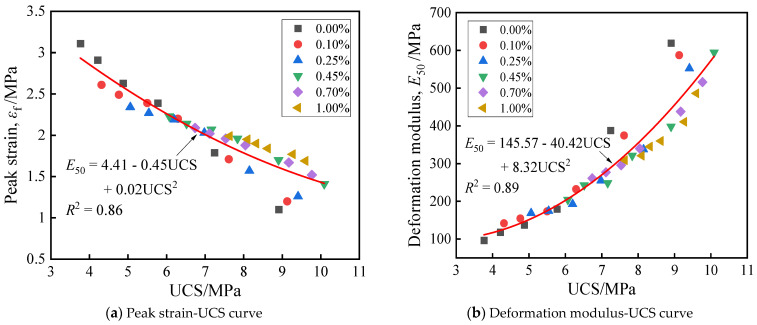
Relationship curves of mechanical parameters of solidified STS specimens.

Figure 12 reveals the intrinsic evolutionary relationships among various mechanical indicators of solidified STS. Significant fitting relationships exist between the peak strain, deformation modulus, and UCS of the specimens: specifically, the peak strain exhibits a pronounced negative correlation with UCS, while the deformation modulus shows a positive correlation with UCS. This phenomenon indicates that as the cementation strength increases, the stiffness of the specimens correspondingly enhances, whereas the plastic deformation capacity at failure relatively diminishes. It is noteworthy that with the further increase in UCS values, the correlation between the peak strain, deformation modulus, and strength exhibits a gradually attenuating trend. Within the range of higher UCS values, the degree of dispersion of data points increases significantly. This suggests that at high strength levels, the mechanical response of the solidification system is not only governed by the total amount of cementitious products but is also influenced by multiple factors, including the uncertainty of fiber spatial distribution and the complexity of the micro-pore structure, leading to greater randomness in the deformation parameters.

The observed relationships between peak strain, deformation modulus, and UCS have practical implications for engineering applications. First, the negative correlation between peak strain and UCS indicates that higher-strength specimens are more brittle, which must be considered in design to avoid sudden failure. Second, the positive correlation between deformation modulus and UCS suggests that strength improvement directly enhances stiffness, beneficial for settlement control but requiring careful attention to cracking potential. Third, the increasing scatter at higher UCS values implies that for high-strength mixtures, the mechanical response becomes less predictable; therefore, a conservative safety factor may be advisable. These relationships can guide the selection of BF content based on desired performance targets (e.g., ductility vs. strength) and support quality control during production by using UCS as an indirect indicator of deformation behavior.

### 4.3. Limitations and Future Research Directions

While this study provides valuable insights into the performance of GCCM- and BF-solidified STS under D-W cycles, several limitations should be acknowledged. First, the experiments were conducted under laboratory conditions with controlled temperature and moisture regimes, which may not fully replicate the complex field environment (e.g., fluctuating groundwater levels, combined chemical erosion, mechanical loading). Second, the specimen size (standard cylindrical samples) may introduce scale effects, and the mechanical response of larger-scale elements or field applications could differ. Third, although BF was mixed according to a standardized procedure, some degree of fiber dispersion variability is inevitable, which could influence the reproducibility of the reinforcement effect. Fourth, the binder content (20% by dry mass of STS) was fixed in this study; the influence of varying GCCM dosage on long-term durability remains to be explored.

Future research should focus on the following aspects: (1) large-scale model tests or field trials to validate the laboratory findings under real service conditions; (2) optimization of fiber dispersion techniques to minimize variability; (3) investigation of the coupled effects of D-W cycles with other environmental factors such as freeze–thaw, acid attack, or carbonation; (4) life-cycle assessment and cost–benefit analysis of the GCCM-BF system for practical engineering applications; and (5) development of predictive models for long-term performance degradation based on accelerated testing.

## 5. Conclusions

By integrating UCS tests, P-wave tests, and laboratory D-W cycle tests, this study systematically investigated the macroscopic mechanical property evolution of STS solidified with BF reinforced GCCM composite binder. Meanwhile, using SEM micromorphology observation and industrial CT 3D reconstruction technology, quantitative characterization and qualitative analysis were conducted on the pore characteristics, crack development laws, and mineral loss process within the solidification system from a multi-scale perspective. The main conclusions are as follows:

(1) The development of the solidification agent GCCM by partially substituting OPC with supplementary cementitious materials can significantly enhance the bearing capacity of STS. The introduction of BF further optimized the solidification performance. At a BF content of 0.45%, the UCS reached a peak value of 10.09 MPa, which was 13.24% higher than that of the unreinforced specimens. In addition, BF significantly improved the deformation compatibility of the material, transforming the failure mode of the specimens from brittle shear to ductile tension-shear combined failure.
(2) The mechanical indicators of solidified STS specimens exhibited significant stage-dependent deterioration characteristics with an increasing number of D-W cycles, with damage accumulating rapidly within the first 5 cycles. The non-uniform swelling–shrinkage stress induced by water migration and physical scouring led to the initiation and propagation of micro-pores and micro-cracks. However, the presence of BF effectively delayed the decline rate of the mechanical property indicators of solidified STS specimens, demonstrating excellent environmental adaptability.
(3) Industrial CT 3D reconstruction and SEM observations confirmed that BF, through its “bridging effect” across micro-cracks and “pull-out energy dissipation mechanism” under compression, pinned the crack tip propagation at the microscopic level and curtailed the evolution of micro-defects into macro-cracks. Quantitative analysis showed that the introduction of BF significantly reduced the *V*_k_ and *V*_l_ of damaged specimens. As the BF content increased from 0.00% to 1.00%, the reductions in *V*_k_ and *V*_l_ of specimens after 8 D-W cycles were 54.32% and 63.23%, respectively.
(4) The hydrophilic minerals such as kaolinite and montmorillonite abundantly present within STS are the intrinsic driving force for damage evolution. During D-W cycles, the crystalline swelling force and osmotic pressure induced by water migration generated non-uniform swelling–shrinkage stress inside the matrix, resulting in the scouring loss of cementitious materials and the initiation of micro-fissures. The three-dimensional random reinforcement network constructed by BF counteracted this swelling–shrinkage strain through physical constraint, significantly enhancing the micro-skeletal stability of the solidification system under extreme moisture field fluctuations.
(5) The optimal BF content for GCCM-solidified STS depends on the service environment. Under static conditions without D-W cycles, 0.45% BF provides the highest initial strength. However, under prolonged cyclic moisture fluctuations, a slightly higher fiber content (e.g., 0.70–1.00%) is recommended to achieve better residual strength and long-term durability. This finding highlights the importance of selecting fiber dosage based on anticipated environmental exposure, providing practical guidance for engineering applications of STS solidification.

## Figures and Tables

**Table 1:** Basic physical and mechanical properties of undisturbed STS.

Parameters	Values
Natural moisture content *w*/%	16.31
Dry density *ρ*_d_/(g·cm^−3^)	1.81
Liquid limit *w*_L_/%	26.82
Plastic limit *w*_P_/%	18.32
Plasticity index *I*_P_	8.50
Optimum moisture content *w*_op_/%	11.21
Maximum dry density *ρ*_dmax_/(g·cm^−3^)	1.37
pH value (solid–liquid ratio 1:1)	8.92

**Table 2:** Chemical composition of raw materials used in GCCM (wt%).

Material	SiO_2_	Al_2_O_3_	Fe_2_O_3_	CaO	MgO	SO_3_	Na_2_O	LOI
OPC	21.50	5.20	3.80	63.20	2.10	2.50	0.20	1.50
GGBS	34.20	16.50	0.80	38.50	7.20	0.50	0.30	0.80
FA	52.80	28.50	6.20	4.50	1.20	0.80	0.60	2.50
FGDG	1.20	0.40	0.20	32.50	0.80	44.50	0.10	20.00
CAP	0.50	70.00	0.30	25.00	0.20	0.10	0.10	3.50

LOI: Loss on ignition.

**Table 3:** Major clinker phase composition of OPC (wt%).

Phase	C_3_S	C_2_S	C_3_A	C_4_AF
Content	52.50	22.00	8.50	10.50

## Data Availability

The original contributions presented in this study are included in the article. Further inquiries can be directed to the corresponding author.

## References

[1] Zhang H., Liu T., Wang W., Cui Y., Wu Y., Qiu X. (2025). Effective curing of waste mud from slurry shield tunnels using OPC-MCA: Experimental investigations and microstructural analysis. Tunn. Undergr. Space Technol..

[2] Zhang C., Yang J., Xie Y., Fu J., Wang S., Yin J. (2022). Utilization of tunnel spoils as a lightweight filling material for the voids behind tunnel excavation contour. J. Clean. Prod..

[3] Alnuaim A., Al-Mahbashi A.M., Dafalla M. (2022). Utilizing tunnel boring machine (TBM)-crushed limestone as a construction material. Materials.

[4] Dinçer A.E., Demir A., Öztürk Ş., Yilmaz K. (2025). A sustainable decision-making framework to evaluate land and seaside disposal options for tunnel spoil: A case study of Trabzon. J. Mt. Sci..

[5] Zhang H., Liu T., Cui Y., Wang W., Qing C. (2025). Experimental study on the performance of basalt fiber combined with cement-based material solidified shield waste mud under the coupled effects of acid corrosion and dry-wet cycles. Constr. Build. Mater..

[6] Choobbasti A.J., Samakoosh M.A., Kutanaei S.S. (2019). Mechanical properties soil stabilized with nano calcium carbonate and reinforced with carpet waste fibers. Constr. Build. Mater..

[7] Durczak K., Pyzalski M., Pilarski K., Brylewski T., Sujak A. (2021). The Effect of Liquid Slurry-Enhanced Corrosion on the Phase Composition of Selected Portland Cement Pastes. Materials.

[8] Guo L., Wu Y.Y., Xu F., Song X.T., Ye J.Y., Duan P., Zhang Z.H. (2020). Sulfate resistance of hybrid fiber reinforced metakaolin geopolymer composites. Compos. B Eng..

[9] Inkham R., Kijjanapanich V., Huttagosol P., Kijjanapanich P. (2019). Low-cost alkaline substances for the chemical stabilization of cadmium-contaminated soils. J. Environ. Manag..

[10] Pyzalski M., Brylewski T., Sujak A., Durczak K. (2023). Changes in the Phase Composition of Calcium Aluminoferrites Based on the Synthesis Condition and Al_2_O_3_/Fe_2_O_3_ Molar Ratio. Materials.

[11] Bedia J., Muelas-Ramos V., Peñas-Garzón M., Gómez-Avilés A., Rodríguez J.J., Belver C. (2019). A Review on the Synthesis and Characterization of Metal Organic Frameworks for Photocatalytic Water Purification. Catalysts.

[12] Zhang H., Wang W., Qiu X., Zheng J., Liu T. (2024). Mechanical properties of fracture-grouted prefabricated sandstone after thermal-acid coupling treatment: An experimental study. Constr. Build. Mater..

[13] Ahmad J., Kontoleon K.J., Majdi A., Naqash M.T., Deifalla A.F., Ben Kahla N., Isleem H.F., Qaidi S.M. (2022). A comprehensive review on the ground granulated blast furnace slag (GGBS) in concrete production. Sustainability.

[14] Renjith R., Robert D., Setunge S., Costa S., Mohajerani A. (2021). Optimization of fly ash based soil stabilization using secondary admixtures for sustainable road construction. J. Clean. Prod..

[15] Mathapati M., Amate K., Prasad C.D., Jayavardhana M., Raju T.H. (2022). A review on fly ash utilization. Mater. Today Proc..

[16] Kolhe S.S., Chang T.P., Chen C.T., Shih J.Y. (2022). Potential application of thermally treated calcium carbide residue as solid CaO activator for No-cement slag-FGDG composite. Constr. Build. Mater..

[17] Zapata J.F., Azevedo A., Fontes C., Monteiro S.N., Colorado H.A. (2022). Environmental impact and sustainability of calcium aluminate cements. Sustainability.

[18] Anburuvel A. (2024). The engineering behind soil stabilization with additives: A state-of-the-art review. Geotech. Geol. Eng..

[19] Zada U., Jamal A., Iqbal M., Eldin S.M., Almoshaogeh M., Bekkouche S.R., Almuaythir S. (2023). Recent advances in expansive soil stabilization using admixtures: Current challenges and opportunities. Case Stud. Constr. Mater..

[20] Xu X.T., Shao L.J., Huang J.B., Xu X., Liu D.Q., Xian Z.X., Jian W.B. (2021). Effect of wet-dry cycles on shear strength of residual soil. Soils Found..

[21] Zhang H., Liu T., Wang W., Cui Y., Zhang D. (2025). Experimental Study on Multiscale Damage Mechanism of Diorite Under the Effects of High Temperature and Acidic Environment. Rock Mech. Rock Eng..

[22] Ji Z., Chen K., Chen J. (2025). Research on strength characteristics and mechanism of cement stabilized phosphogypsum materials under dry and wet cycles. Sci. Rep..

[23] Li T., Yang Z., Ma D., Tian J., Feng H. (2025). Effects of dry–wet cycles on the dynamic characteristics of cement-stabilized loess. Bull. Eng. Geol. Environ..

[24] Akbari H.R., Sharafi H., Goodarzi A.R. (2021). Effect of polypropylene fiber and nano-zeolite on stabilized soft soil under wet-dry cycles. Geotext. Geomembr..

[25] Zhang H., Liu T., Cui Y., Wang Z., Wang W., Zheng J. (2024). Compression and shear properties of OPC-MCA and basalt fiber cured shield waste mud after dry-wet cycles. Constr. Build. Mater..

[26] Jagadeesh P., Rangappa S.M., Siengchin S. (2024). Basalt fibers: An environmentally acceptable and sustainable green material for polymer composites. Constr. Build. Mater..

[27] Kim S.H., Lee J.H., Kim J.W., Lee S.Y., Park S.J. (2022). Interfacial behaviors of basalt fiber-reinforced polymeric composites: A short review. Adv. Fiber Mater..

[28] Khandelwal S., Rhee K.Y. (2020). Recent advances in basalt-fiber-reinforced composites: Tailoring the fiber-matrix interface. Compos. B Eng..

[29] (2007). Standard for Engineering Classification of Soil.

[30] Liu J., Wen S., Jing B., Liu W. (2022). Effect of dry–wet cycles on the shear strength of weathered red sandstone soil. Soil Mech. Found. Eng..

[31] Huang G., Zheng M. (2021). Effect of Dry-Wet Cycling on the Residual Strength Characteristics of Coal Measure Soil. KSCE J. Civ. Eng..

[32] Xu J., Li Y., Wang S., Wang Q., Ding J. (2020). Shear strength and mesoscopic character of undisturbed loess with sodium sulfate after dry-wet cycling. Bull. Eng. Geol. Environ..

[33] Gao C., Du G., Guo Q., Zhuang Z. (2020). Static and Dynamic Behaviors of Basalt Fiber Reinforced Cement-Soil after Freeze-Thaw Cycle. KSCE J. Civ. Eng..

[34] Shu Y., Zhang J. (2023). Effect of Basalt Fiber Content and Length on the Strength and Crack Development of Polyvinyl Alcohol/Basalt Hybrid Fiber-Reinforced Cement Soil. Polymers.

[35] Chen J., Mu J., Chen A., Long Y., Zhang Y., Zou J. (2024). Experimental Study on the Properties of Basalt Fiber–Cement-Stabilized Expansive Soil. Sustainability.

[36] Zhang H., Liu T., Cui Y., Zheng J., Wang W., Li Y. (2024). Experimental study on the deterioration mechanisms of physical and mechanical properties of red sandstone after thermal-acid coupling treatment. Constr. Build. Mater..

[37] Liu X., Han M., Liu T., Liu L. (2023). Macroscopic and Microscopic Characteristics of Strength Degradation of Silty Soil Improved by Regenerated Polyester Fibers under Dry–Wet Cycling. Polymers.

[38] Wei X., Yu J., Dai G., Liu L., Dou L. (2023). Fiber for recycled aggregate concrete reinforcement: A review. J. Text. Inst..

[39] Ji H., Fan X., Ding F. (2025). Optimization of Synergy Among Granulated Blast Furnace Slag, Magnesium Oxide, and Basalt Fiber for the Solidification of Soft Clay. Materials.

[40] Li L., Liu W., Fei X., Cheng Z., Li W. (2026). Coconut shell fiber for reinforcing lime-stabilized soil: A sustainable approach to improve resilience. Can. Geotech. J..

[41] Guo Z., Cao X., Wu J., Zhong Y., Liu Q. (2026). Experimental study on the disintegration characteristics of rare earth tailings improved by BF-MICP. Sci. Rep..

[42] Zhang H., Liu T., Cui Y., Wang W., Yang X., Huang X. (2024). Strength deterioration and damage mechanism of grout-reinforced fractured sandstone under the coupled effects of acidic erosion and freeze-thaw cycles. Constr. Build. Mater..

[43] Sun L., Tanskanen J.T., Hirvi J.T., Kasa S., Schatz T., Pakkanen T.A. (2015). Molecular dynamics study of montmorillonite crystalline swelling: Roles of interlayer cation species and water content. Chem. Phys..

